# Influence of a periodized circuit training protocol on intermuscular adipose tissue of patients with knee osteoarthritis: protocol for a randomized controlled trial

**DOI:** 10.1186/s12891-018-2325-y

**Published:** 2018-11-30

**Authors:** Aline Castilho de Almeida, Maria Gabriela Pedroso, Jessica Bianca Aily, Glaucia Helena Gonçalves, Carlos Marcelo Pastre, Stela Marcia Mattiello

**Affiliations:** 1 Department of Physical Therapy, Federal University of São Carlos (UFSCar), Km 235,Rod. Washington Luís–SP310, Postal code, São Carlos, (SP) 13565-905 Brazil; 2 Department of Physical Therapy, São Paulo State University (UNESP) - School of Sciences and Technology, 305, Roberto Simonsen St., Presidente Prudente, (SP) 19060-900 Brazil

**Keywords:** Intermuscular fat, Circuit workouts, Adipokines, Quadriceps, Inflammation, CT scan, Physical therapy, Rehabilitation

## Abstract

**Background:**

The objective of this study is to analyze the influence of a 14-week periodized circuit training protocol on patients with knee osteoarthritis (OA), in randomized intervention groups, on thigh intermuscular adipose tissue (interMAT), body composition, systemic inflammation, cartilage degradation, and its repercussion on pain, functional performance and quality of life.

**Methods:**

This study presents a protocol for a randomized controlled trial. Sixty selected participants diagnosed with knee OA grades II and III, 40–65 years old and BMI < 30 kg/m^2,^ will be randomly divided into three groups:periodized circuit training, strength training, and educational protocol. The circuit training and strength training protocols consist of 14-week training protocols conducted 3 times a week. The circuit training group will perform selected exercises previously stratified as light, moderate, and intense, arranged progressively in a circuit model, the strength group will perform regular strength exercises, and the educational protocol group will participate in a 14-week protocol with lectures twice a month about healthy lifestyles. Baseline and follow-up evaluations will be conducted for thigh interMAT (computed tomography), body composition (DXA), inflammation (IL-1β, IL-6, IL-10, TNF-α, leptin, and adiponectin), and joint degradation biomarkers (uCTX-II and sCOMP), performance-based tests (30s Chair Stand Test, 40 m Fast-paced Walk Test and Stair Climb Test), quadriceps and hamstring maximal isometric voluntary contraction (MIVC), and questionnaires (WOMAC and pain catastrophizing scale). Repeated measures ANOVA will be used to compare differences between groups (circuit training X strength training X educational protocol) at the different times of assessment (baseline x follow-up or baseline x during protocol x follow-up) for each of the dependent variables. When significant main effects were found, the pots hoc Bonferroni test will be used to identify statistical differences. A significance level of 5% (*p* < 0.05) will be adopted.

**Discussion:**

This will be the first randomized controlled trial to assess the effects of a circuit training protocol on patients with knee OA on thigh intermuscular adipose tissue (interMAT). Given the prevalence and impact of OA and the widespread availability of this intervention, assessing the efficacy of a low-cost, non-pharmacological, and non-invasive treatment for knee OA patients has the potential for immediate and high clinical impact.

**Trial registration:**

ClinicalTrials.gov, NCT02761590, registered in May 4, 2016.

**Electronic supplementary material:**

The online version of this article (10.1186/s12891-018-2325-y) contains supplementary material, which is available to authorized users.

## Background

Knee osteoarthritis (OA) is a chronic inflammatory degenerative joint disease that commonly causes pain and limits activity, placing a significant burden on healthcare services [1–3]. Changes in body composition such as a decrease in muscle mass associated with an increase in adipose tissue are characteristics that can contribute to OA progression. Moreover, fat infiltration can change the orientation of muscle fibers, thereby reducing the power production capacity [4, 5].

Higher amounts of intermuscular adipose tissue (interMAT) in the thighs of patients with knee OA has been considered clinically significant due to the negative associations with knee extensor strength, physical function, and the systemic inflammatory process [6–8]. InterMAT can be considered a deposit of ectopic fat similar to visceral adipose tissue, which can release proinflammatory cytokines [9].

Modulation of the inflammation present in OA has been related to the occurrence of some cytokines, especially interleukin-1β (IL-1β), interleukin-6 (IL-6), and tumor necrosis factor-α (TNF-α), which are critical mediators of metabolic disturbance and increased catabolism of joint tissues [10], and may lead to progressive loss of muscle mass [11] and interfere with cartilage degradation [12]. Adipocytes release adipokines such as leptin and adiponectin, which can cause and exacerbate chronic low-level systemic inflammation. Studies suggest that leptin may act to regulate chondrocyte metabolism and has been related to the metabolic (non-mechanical load) effect of obesity on joint disease and could explain the association between joint disease and metabolic syndrome disorders [10, 13, 14]. Adiponectin has also been proposed as a systemic biomarker of OA. Plasma adiponectin was significantly higher in a population of OA patients [11]. In muscle, adiponectin has been shown to increase fatty acid oxidation and glucose uptake, and to attenuate local inflammation [15].

The catabolic processes of articular cartilage evaluation present in OA are very important for better understanding of its pathogenesis, as well as being a method to evaluate its development in the short term. Biomarkers, such as uCTX-II and COMP, have the capacity to detect early joint degradation in degenerative diseases such as OA [16], being, therefore, significantly associated with the incidence and progression of OA [17].

Although a number of biochemical markers of joint tissue turnover have been developed and tested, the majority of studies have been limited to cross-sectional studies of people with and without OA or observational studies of OA progression [18]. It has been described in the literature that physical exercise modifies the inflammatory condition of the body, especially by modifying the circulation of IL-6 [19] and increasing anti-inflammatory cytokines such as IL-10 [20]. Thus, these cytokines are related to basic morbidities under the influence of different types of exercises.

Considering the reduction in physical activity and quality of life found in individuals with knee OA (due to the influence of inflammation and changes in body composition) [21], non-pharmacological, conservative clinical guidelines are encouraged, and physical exercises are recommended due to their beneficial effects, low potential for adverse effects, and low cost [22–24].

Studies assessing the effects of exercise on knee OA are widespread and are intended to reduce pain, and improve muscle strength, stability of joints, and aerobic fitness, leading to improvement in functionality [24, 25]. Although several studies have demonstrated its effectiveness in increasing strength, reducing pain, and improving functionality [26], there is no evidence of the effect of exercise on thigh composition (adipose tissue and muscle mass) and the related impairment in individuals with knee OA.

It is known that physical exercise can increase muscle mass and muscle strength [27] and has shown promise in protecting the knee joint and reducing inflammatory cytokines [28, 29]. In a systematic review, Juhl et al. (2014) investigated training protocols used to treat patients with knee OA and reported that optimal exercise programs for this population should focus on improving aerobic capacity, quadriceps strength, and lower limb performance [25]. However, Bennell and Hinman (2011) reported that a combination of both resistive and aerobic training is better suited to deal with the various complications associated with OA [23]. Similarly, Nguyen et al. (2016), in a review study related to the efficacy and safety of exercise therapy and strength training, reported that although rehabilitation is a key treatment in OA and widely recommended, the ideal method for therapy programs remains inconsistent [30]. In this way, enriching the reasons for exercise prescription seems to be a gap that needs filling.

One way to combine the benefits of these modalities is circuit training, used to stimulate both systems, which promotes cardiovascular and muscle strengthening [31, 32]. The effects of circuit training on several chronic disorders are known and positive effects have been shown on body composition by reducing body fat, which can be achieved by a repeated series of exercises, with little or no rest interval between them [33]. This type of exercise can more effectively activate lipolysis of adipose tissue than conventional aerobic training [31, 34]. This allows many people to participate in the same training session due to low total duration of the exercises [31, 33, 35, 36], which promotes high retention and adherence of participants [37].

Despite having recently attracted the interest of researchers due to the potential benefits that this technique provides, the effects of a periodized circuit training protocol on interMAT remain unclear. Only three studies in the consulted literature have evaluated the effects of circuit training on knee OA patients. These studies showed a reduction in body mass and adipose tissue (assessed by dual X-Ray absorptiometer (DXA)), increase in muscle strength, improved knee function, and a reduction in pain compared with a control condition [38–40]. However, the training protocol did not follow a periodized model.

Adipose tissue concentration and muscle quality are directly linked with physical activity level and exercise intensity. Few studies have explored the physical activity level of their sample [41–43] and used it to normalize or discuss data. In older adults, a greater physical activity level is associated with less visceral adipose tissue, subcutaneous fat, and interMAT [44].

To date, no studies have assessed the changes in muscle fatty infiltration in the thigh after exercise and its implications, such as inflammation levels in the knee OA population. There is evidence that exercise in combination with diet is effective for decreasing total body fat mass, inflammation levels, and pain, in addition to improving physical function in overweight and obese adults with knee OA [45]. In overweight and obese adults, resistance training improved body composition, including reduction in thigh interMAT [46]. Meanwhile, in older fallers, there were no significant changes in thigh interMAT content after 3 months of resistance training [47]. It seems that exercise can decrease fat infiltration in thigh muscles of overweight and obese. However, in older fallers this was not seen.

Considering the possible alterations in muscle function and morphology caused by the presence of fat tissue in thigh muscles, it is essential that new study proposals involving a periodized circuit training protocols identify not only body fat reduction, but also the local effects of the amount of interMAT.

It is understood that a randomized controlled trial, the gold standard in intervention studies [48], is the best way to evaluate new treatments for a given variable, as suggested by Messier et al. (2015) in the study recommendations, design, and conduct of clinical trials in patients with OA [49]. The present study protocol has an investigative character and we emphasize its originality, mainly because the influence of periodized circuit training on the main study variable (interMAT) is still unknown.

The results of this research may contribute to elaboration of future treatment strategies and early intervention in the causative and potentiating factors of the disease, verifying the effects of a low-cost, non-pharmacological, and non-invasive treatment to provide subsidies for better rehabilitation planning for patients with knee OA.

## Methods/Design

### Aim

The main objective of the present study will be to compare the influence of 14-weeks of periodized circuit training with strength training and an educational protocol on thigh interMAT and body composition. Secondarily, the present study will investigate these effects on indicators of cartilage degradation and systemic inflammation, and its repercussions on physical function, pain, and quality of life in patients with knee OA.

Our general hypothesis is that both circuit training and strength training protocols will promote decreases in thigh interMAT, associated with improvement in body composition and muscle strength, and a reduction in inflammatory cytokine concentration (IL1β, IL6, TNF-α), pain, and stiffness in patients with knee OA, leading to consequent improvement in quality of life, compared to the educational protocol. However, it is hypothesized that these benefits, mainly related to the improvement in the body composition and reduction in interMAT will be more evident after the circuit training, compared to the strength training. Additionally, it is also hypothesized that biomarkers of joint degradation may signal exercise efficiency for both groups over the application of both training protocols.

The following methodology is in full agreement with the SPIRIT (Standard Protocol Items for Randomized Trials) recommendations [50–52] [50–52] and the Osteoarthritis Research Society International (OARSI) recommendations for RCTs [53] to ensure methodological rigor. Consolidated Standards of Reporting Trials (CONSORT) [54] guidelines will be followed for reporting the results in a subsequent article.

### Study design

The present proposal is characterized as a single-blinded randomized controlled trial design. This is the first version of the study protocol, submitted to the registry of clinical trials *Clinical Trials* (clinicaltrials.gov) and registered in May 4, 2016, under identification code NCT02761590.

### Ethical aspects

This study was approved by the Research in Human Ethics Committee (CEP) of the Federal University of São Carlos-SP. Participants will be informed about the procedures that will be performed throughout the research, and after agreeing to participate, will sign an informed consent form (ICF). This study will be conducted according to Resolution 466/12 norms of the National Health Council on research involving humans.

### Blinding

The outcome assessor will be blind to group allocation and will not be involved in the interventions or attend any of the testing. The physical therapists supervising the exercise intervention sessions will not be blinded. The statistician will be blinded to group allocation prior to completion of the statistical analysis. With training and standard operating procedures, it is anticipated that any performance bias due to lack of blinding will be minimized.

### Recruitment

Participants from the community of São Carlos, Brazil, will be invited to participate in this study. Three main methods will be used to recruit potential participants: advertisements will be placed in local newspapers, magazines, and social media. Potential participants will be invited to complete a phone interview to pre-screen their eligibility to ensure that they meet the study selection criteria prior to randomization. If deemed eligible, an appointment will be scheduled to confirm eligibility, sign the ICF, and complete baseline evaluations.

### Sample

To determine the sample size, the data of thigh interMAT from the study of Griessmann et al. (2014) were considered [55]. 16 participants per group was stipulated through a two-tailed hypothesis test, with a 5% level of significance and 80% test power.

Considering the possible sample loss in the course of the study, 20 subjects each, totaling 60 participants of either sex will be included in this study.

To be eligible, participants will be required to fulfill the following criteria:

i. Aged between 40 and 65 years;
ii. KOA clinically diagnosed fulfilling the American College of Rheumatology classification criteria of knee pain on most days of the past month and radiographically, classified as grades 2 or 3 according to Kellgren and Lawrence criteria [56].
iii. Knee pain for ≥3 months;
iv. Overall average knee pain in the last week ≥4 on a 0 to 10 Visual Analogue Scale (VAS).

The exclusion criteria are:

i. Knee surgery;
ii. Body mass index (BMI) ≥30 kg/m^2^;
iii. Previous history of lower limb trauma;
iv. Physical therapy, chiropractic, or acupuncture treatment or exercises specifically for the knee within the previous 6 months;
v. Walking more than 30 min continuously daily or participating in a regular (more than twice a week) exercise program;
vi. Current or past (within 3 months) oral or intra-articular corticosteroid use;
vii. Systemic arthritic conditions such as rheumatoid arthritis;
viii. Inability to walk unaided as this is necessary for some of the physical testing;
ix. Medical condition precluding safe exercise such as uncontrolled hypertension, heart condition, cardiac pacemaker use, chronic diseases, impaired renal function, or any other medical condition that precludes participation in the study [57, 58];
x. History of severe muscular lesions (above grade I);
xi. Motor impairment due to neuromuscular diseases;
xii. Cognitive deficits that compromise understanding of the tests;
xiii. Starting any other exercise protocol or physical therapy during the study.
xiv. Unable to comply with the protocol such as inability to attend therapy session or attend assessment appointment at the University.
xv. Kellgren and Lawrence grades 0, 1, and 4.

### Procedures

**Fig. 1:**
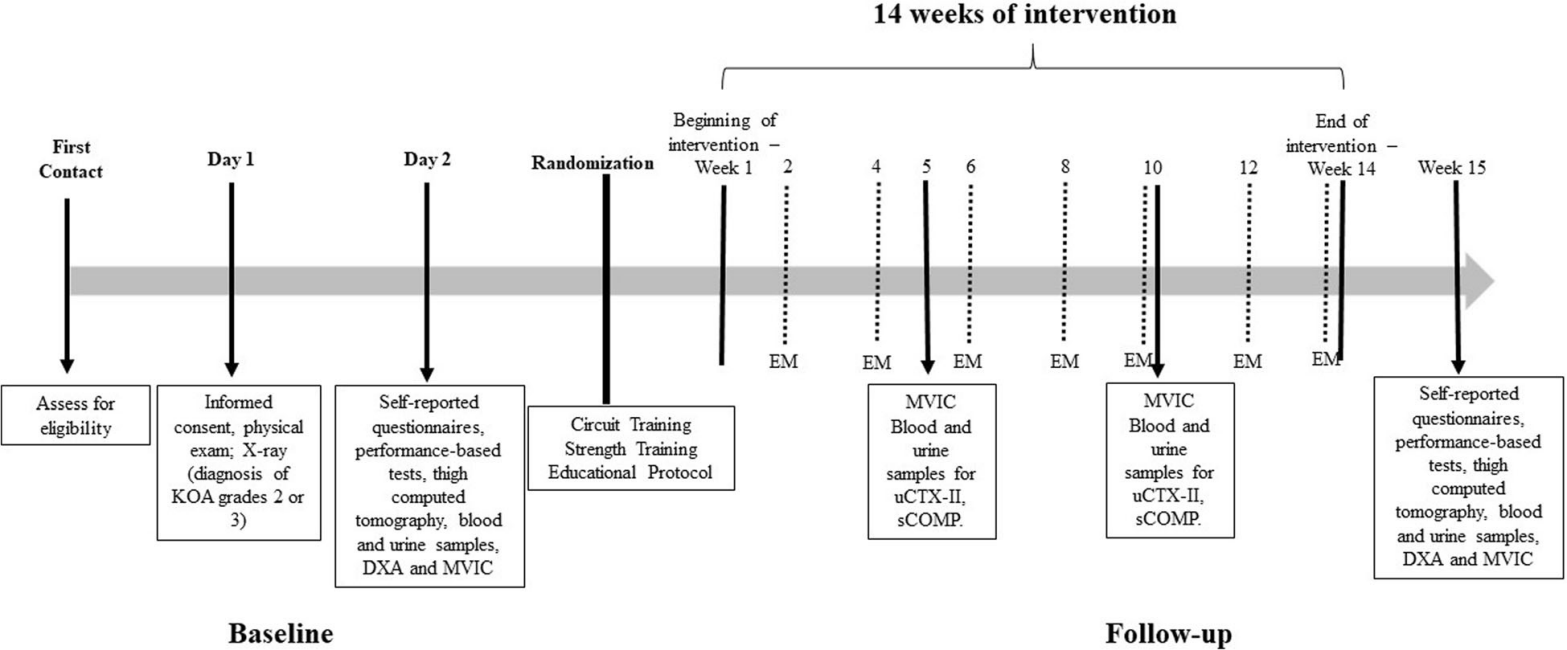
Timeline of study procedure; EM: Educational meeting sessions; DXA: dual X-Ray absorptiometry; MVIC: maximal voluntary isometric contraction

All experimental procedures will be performed following the timeline shown in Fig. 1. The evaluations will be conducted at the Articular Function Analysis Laboratory (LAFAr) and the training protocols will be conducted at the Physical Therapy Department of the Federal University of São Carlos (UFSCar), in a room with a controlled temperature (between 21 °C and 23 °C).

On day 1, participants will be guided and familiarized with the experimental procedures to be performed. An evaluation form (personal and anthropometric data, blood pressure, knee pain, and previous medical history) and the informed consent form will be completed. During the same visit, the body composition test (Dual Energy X-ray Absorptiometry - DXA) will be performed and the participant will receive a referral for the knee radiographic exams.

Day 2 will be scheduled after the OA radiographic diagnosis and classification. During this visit, the WOMAC *(Western Ontario and McMaster Universities)* and Pain Catastrophizing Scale (PCS) questionnaires will be completed. Subsequently, the participants will perform three functional performance-based tests (40 m fast-paced walk test, 30 s chair stand test, and stair climb test), and the strength test*.* The functional performance tests and the strength test will be performed in random order. Participants will then receive the referral and guidelines for the computed tomography exam for thigh composition (adipose tissue and muscle mass) analysis and blood and urine samples will be collected for inflammatory and collagen activity marker analysis, identifying the systemic responses to exercise. After the baseline evaluations (Days 1 and 2), participants will be randomized through a randomization website (https://www.random.org/) into three groups: periodized circuit training, strength training, and educational protocol. The group distribution will be balanced for: number of males and females, age, body mass index (BMI), and radiographic severity. A researcher not involved with the evaluations and intervention sessions will be in charge of conducting the participant randomization and balance distribution. During and at the end of the 14 weeks intervention protocols, follow-up evaluations will be scheduled (Fig. 1).

Data collections will be performed over the 14 weeks of the study. At baseline and post 14 weeks intervention (follow up) the following will be performed: computed tomography of thighs, maximal voluntary isometric contraction (MVIC), performance-based tests, blood and urine samples, questionnaires, and DXA. In the 5th, 10th, and 14th weeks, blood and urine samples will be collected for sCOMP and uCTX-II analysis and the MVIC test will be conducted.

All analyzes will be performed for all groups blindly, i.e., the evaluator of the collected data will be blinded to the group allocation.

### X-ray

All participants will undergo radiographic exams of the knees for the diagnosis of unilateral or bilateral osteoarthritis. Bilateral posterior-anterior weight-bearing semi flexed knee x-rays will be used to identify tibiofemoral OA and skyline views to identify patellofemoral OA [59, 60]. The Kellgren and Lawrence (1956) [56] and American College of Rheumatology criteria will be considered to diagnose and classify KOA, considered as: Grade 0, without changes; Grade I, minimal osteophytes with dubious presence; Grade II/o, defined osteophytes and absence of joint space decrease; Grade II, defined osteophytes and minimal reduction in joint space, grade I in the classification of joint space reduction of Altman and Gold (2007); Grade III, presence of osteophytes and moderate reduction in joint space; Grade IV, significant reduction in joint space and subchondral bone sclerosis [61, 62].

### Thigh computed tomography

Intermuscular adipose tissue of both thighs will be the primary outcome for this study. For this, computed tomography scans will be conducted using a Multislice Tomograph (Brilliance CT 16-slice, Phillips), located at the University Hospital of the Federal University of São Carlos. The exam will be performed by a specialized radiologist.

Image acquisition will be performed as described by Messier et al. (2013) [2]. Participants will be placed supine with their legs held in a neutral position. A 2-dimensional topogram will be obtained from the pelvis to the knees. In order to obtain the scans, the following parameters will be established: helical mode, 120 KV, 150 mAs, with reconstruction of both legs at 5 mm slice thickness and 50 cm display field of view (DFOV).

To establish the site of interest for analysis of thigh composition, the total femur length (from the greater femoral tuberosity to the lower border of the medial femoral condyle) will be transected into three parts. The junction between the proximal and mid-third will be marked for measurement. The area analyzes of thigh composition (interMAT, subcutaneous adipose tissue, and muscle mass) will be manually performed using ITK-SNAP (version 3.6) software, and the area of interest will be selected by a scan according to tissue density attenuation rates for quantification of adipose tissue presented in cm^2^. Additionally, a manual line will be made separating the bony part of the soft tissues, and then the adipose tissue present within the bone area will be subtracted from the area obtained by measuring the interMAT and subcutaneous adipose tissue. Skeletal muscle and adipose tissue areas will be calculated by the range of attenuation values for skeletal muscle (0 to 100 HU) and adipose (− 190 to − 30 HU) tissue [63, 64]. Test-retest reliability on thigh scans re-analyzed 1 week apart was ICC = 0.973 and between evaluators was ICC = 0.986 (*n* = 20).

### Body composition

Dual Energy X-Ray Absorptiometry (DXA, Hologic Discovery A, Bendford, MA) will be used for measurements including lean body mass, body fat mass, and bone mineral density, allowing, therefore, estimation of the total body composition and per body segment.

The scanning will be performed according to the manufacturer’s recommendations. According to the instruction manual, the operator should check that no metal or plastic objects remain in the scanning area, since it may alter the attenuation ratios of the DXA energy [65]. This includes hair clips and pins, snaps, zippers and buttons, jewelry, earrings, bracelets, watches, or rings. The participants will be asked to arrive in a fasted state (at least 4 h) and not to perform any physical activity for 24 h before the exam in order to reduce the biological variability [66].

The participant will be placed in the supine position, in the center of the scanning table, with the head just below the head line marked on the table and remain unmoving during the examination. DXA software will automatically define areas of regional body estimates (left and right arms, legs and trunk) [65]. For this study, it was defined that segment and total body composition areas will be considered to analysis.

### Inflammatory and degradation of articular cartilage biomarkers

Blood samples will be collected in the early morning after a 12-h fast. In total, 4 ml of blood will be collected from the antecubital vein of each participant using a standard procedure heparin-coated vacutainer tube by a specialized laboratory. Participants will be asked to discontinue the use of anti-inflammatories for 72 h prior to collection.

After collection, the blood will remain for 4 h at rest in the refrigerator. Subsequently it will be centrifuged at 1500 rpm for 10 min and the supernatant will be stored as aliquots and frozen at − 80 °C. Serum concentrations of IL-1-β, IL-6, TNF-α, IL-10, leptin, adiponectin, and sCOMP will be measured by the ELISA method (Enzyme-Linked Immunosorbent Assay), according to the manufacturer’s recommendations (Quantikine, R&D Systems, Minneapolis, MN). Patients will be instructed to perform the blood collections in the morning and remain at rest for 30 min [67].

To assess uCTX-II concentration, participants will be instructed not to perform physical activities for 24 h prior to collection, as well as which, urine will be collected in two specific containers (each with a capacity of 80 ml) of the first urine of the day. Participants will be instructed to fill most of the containers provided and keep the containers in the refrigerator (2 to 8 °C) until delivery to the researcher, which should be no more than 2 h after collection. One of the samples will be sent to the laboratory responsible for determining the creatinine concentration, while the other sample will be centrifuged at 3000 rpm for 10 min for homogenization and stored in cryovials for freezing at − 80 °C in a freezer. The procedures will be performed according to the manufacturer’s instructions (Elabscience Biotechnology, Texas, USA) to detect the uCTX-II concentration by the ELISA method.

Samples will be read in a spectrophotometer specific for microplate reading, using a 490 nm filter. The uCTX-II concentration values (ng/L) will be normalized by the total urine creatinine (mmol/L) as well as the correction of uCTX-II concentration unit in ng/mmol [46, 68].

### Questionnaires

#### WOMAC

The WOMAC (*Western Ontario and McMaster Universities*) questionnaire will be used to assess pain, stiffness, and physical function. This is a self-administered instrument that addresses the impact and restrictions specifically in the quality of life of patients with lower limb OA, translated and validated to Portuguese [69]. The WOMAC contains 24 self-reported issues, based on information within 72 h prior to its application, divided into three domains: pain, stiffness, and physical function. Scoring is performed using a Likert scale, wherein each issue scores between 0 and 100, distributed as follows: 0 = none; 25 = mild; 50 = moderate; 75 = intense; and 100 = very intense [69]. The final score will be determined by the maximum score for each domain; the higher the score, the worse the pain, stiffness, and physical function.

#### Pain catastrophizing scale (PCS)

The Pain Catastrophizing Scale is a self-administered scale, easy and fast to apply. This scale will be used to identify individuals with psychological characters for catastrophizing. This instrument consists of 13 items, in which the patient should report the degree of thought or feeling described in relation to pain, always respecting a graduation of 5 points. The total score is given by the sum of all items, ranging from 0 to 52 points. The patients will be asked to answer the questions according to the thoughts and feelings that develop when they are affected by pain, regardless of whether at the moment of the interview the patient is in pain [70].

### Performance-based tests

The performance-based tests selected for this study follow the OARSI recommendations for specific purposes of functional capacity evaluation of patients with OA of the knee or hip [71].

- 40 m fast-paced walk test (40 m FPWT)

This test will be conducted to evaluate a short distance walking activity and changing direction during walking. A 10 m walkway will be marked out with bright colored tape at each end. A cone will be placed approximately 2 m before the start mark and 2 m beyond the finish mark of the 10 m walkway for turning. Participants will be instructed to wear comfortable walking footwear (e.g. tennis shoes).

Participants will be asked to walk as quickly but as safely as possible, without running, along a 10 m walkway and then turn around a cone, return, and then repeat the walk again for a total distance of 40 m (3 turns). Regular walking aids will be allowed and recorded. Timing will start on the signal to start at the start line and terminate once the participant crosses back over the start line after completing the 40 m (4 × 10 m). When the participant crosses the 10 m mark, timing will be paused whilst the participant turns around the cone and then resumed once they cross the 10 m mark again. The same will be repeated for the following turns and the timer stopped once the participant crosses the start line for the final time. The time of one trial will be recorded to the nearest 100th of a second and expressed as speed m/s by dividing distance (40 m) by time (s) [71].

- 30 s chair stand test (30s-CST)

This test will be conducted to evaluate the sit-to-stand activity, lower body strength, and dynamic balance. This test will evaluate the maximum number of chair stand repetitions possible in a 30 s period. The back of the chair will be placed against a wall, so the chair does not slide backwards. Participants will be instructed to wear comfortable walking footwear (e.g. tennis shoes). The same chair will be used for all participants, at baseline and follow up assessments. A straight back chair will be used with a 44 cm seat height, without arms.

The participants will sit on the chair in a position that allows them to place their feet flat on the floor, shoulder width apart, with knees flexed slightly more than 90 degrees so that their heels are somewhat closer to the chair than the back of their knees. Participants will be instructed to cross their arms at the wrists and hold them close to the chest (across chest). The tester will stand close to the side of the chair for safety and so they can observe the technique, ensuring that the participant reaches a full stand and full sit position during the test. A practice trial on one slow paced repetition will be performed before testing to check technique and understanding. From the sitting position, the participant will stand up completely with hips and knees fully extended, then sit completely back down, so that their bottom is in full contact with the seat. This will be repeated for 30 s. If the person is not able to stand even once then they will be allowed to place their hands on their legs or use their regular mobility aid. This will be then scored as an adapted test score.

On the signal to begin, the stop watch will be started. The total number of chair stands (up and down equaling one stand) completed in 30 s will be counted. If a full stand has been completed at the end of the 30 s (i.e. standing fully erect or on the way down to the sitting position), then this final stand will be counted in the total. The participant will be allowed to stop and rest if they become tired, but the timer will continue. If the person is not able to stand even once then the score for the test will be zero. If the person is able to stand with adaptions, such as hands placed on their legs, then the number of stands will be recorded as an adapted test score and the adaptations made to the test indicated [71, 72].

- Stair climb test (SCT)

This test will be conducted to evaluate ascending and descending stair activity, lower body strength, and balance, using a 12-steps flight of stairs with handrails. Each step will measure 16 cm in height and 30 cm in depth, and adequate lighting, free from traffic and external distractions will be ensured. Participants will be instructed to wear comfortable walking footwear (e.g. tennis shoes), and if safety is of concern, the tester will stand behind/below the participant, going up the stairs and ahead/to the side coming down the stairs. If there is no concern for safety, the tester will remain at the start/finish position on the ground landing. A practice trial with the tester standing in the above positions will be conducted to assess safety.

The participant will be instructed to ascend and descend the flight of stairs as quickly as possible but in a safe manner, using the handrail only if needed (if used, this will be recorded). Timing (stop watch) will begin on the signal to start and terminate when the participant returns with both feet to the ground level. The participant can stop and rest if needed but the timer will continue. The total test duration will be timed (in seconds), with longer times indicating more compromised physical function [72].

### Muscle strength test - maximal voluntary isometric contraction (MVIC)

Knee maximal voluntary isometric contraction (MVIC) extension and flexion will be measured using a *handheld* dynamometer (Lafayette Instruments, Lafayette, IN, USA). Prior to the tests to measure the MVIC, participants will be positioned and asked to perform sub-maximal contractions for warm-up and familiarization with the test. Quadriceps MVIC will be performed in the seated position with a hip angle of 90° flexion and knee angle of 0° (full extension). A Velcro strap (attached to the floor) will be wrapped around the ankle, and another strap will be used to maintain the pelvis against the table. Evaluation of the knee flexor MVIC will be performed at 15° knee flexion in the supine position. A Velcro strap will be wrapped around the ankle and another to secure the pelvis against the table.

The tester will place the dynamometer at the front of the ankle under the Velcro strap to measure knee extension force, and at the back of the ankle under the Velcro strap to measure knee flexion force. Before each test, the tester will demonstrate the direction of the force required. The participant will be instructed to contract “as forcefully as possible”, with a gradual increase in force and strong verbal encouragement will be provided during the contractions. The tester will maintain the dynamometer stable, to prevent it from being displaced. The participants will perform 3 MVIC separated by a 60-s pause (Selistre et al. 2017), and the highest value will be used for analysis. The MVIC will be expressed in kgf [73]. The reliability of the isometric muscle tests with a handheld dynamometer has been reported previously. Studies demonstrated excellent concurrent validity compared with an isokinetic dynamometer and also excellent inter-tester and intra-tester reliabilities for measuring maximal isometric strength in the main movements of lower limbs [74–77]. Additionally, in a pilot study with 10 participants with the same sample characteristics, a two-way mixed model was used to calculate the intraclass correlation coefficient (ICC) within trials. The average measure with 95% confidence interval (CI) and coefficient of variation (CV) were calculated in order to demonstrate the reliability of the mean of recordings. The ICC values of MVIC were higher than 0.80, ranging from 0.88 to 0.98. The CV values ranged from 11 to 32%.

### Muscle quality

Muscle quality is the intrinsic ability of muscle tissue to produce force, which will be estimated through the ratio between the muscle strength, obtained by MVIC, and lean mass of the thigh, obtained by computed tomography (kgf/cm^2^) [78].

### Periodized circuit training protocol (CT)

#### Selected exercises

For elaboration of the periodized circuit training protocol, specific exercises for the knee OA population were selected from previously published studies [79–81].

These exercises were previously arranged in a circuit model: upper body (UB), lower body (LB), and trunk and global exercises (which involve the whole body). A total of 47 exercises were selected (Additional file 1) and then stratified in a pilot study according to the stress intensity levels; light, moderate, and intense. For the stratification, all exercises were performed by 10 subjects with similar characteristics to this study sample (age, demographics, and clinical condition). All participants in the pilot study used a heart rate monitor (Polar V800, Polar Electro Oy, Finland) to obtain HR and the Rating of Perceived Exertion (RPE) was evaluated through the Borg scale with 6–20 points, before and immediately after each exercise. Subsequently, the percentage of the maximum heart rate (%HRmax) was calculated, by subtracting 220 from the participant’s age [82]. For stratification, the Muyor (2013) study was considered, with Rating of Perceived Exertion (RPE) values 6–10: mild; 11–14 moderate; 15–20 intense; and ​​%HRmax values: < 54% light; 55–69% moderate; > 70% intense [82].

The 47 exercises selected were divided into three days (20 per day), with a 48-h interval between them. The participants were instructed to perform each exercise for 30 s, as fast as possible, and a rest interval was given between exercises, considered sufficient for HR to return to pre-exercise values. The interval time ranged from 1 to 3 min. Therefore, with the results of the pilot study, it was possible to organize these exercises through the 14-week training protocol, respecting the intensity progression (light, moderate, and intense).

During the training protocol, it was determined that the exercises stratified as mild will last 20 s, moderate exercises will last 30 s, and the intense exercises for 40 s. Between each station, as described above, there will be a maximum of 30 s rest. This strategy will be used to require the contraction of several muscle groups, with a short rest between stations, causing cardiovascular and metabolic benefits and greater reduction in adipose tissue [83]. It should also be noted that this technique, in addition to resulting in a lower total exercise time, maximizes the impact of an exercise protocol in a short time.

#### Application of the periodized circuit training protocol

The circuit training protocol will be conducted in three sessions per week for 14 weeks, totaling 42 exercise sessions. The minimum percentage of sessions that the participants is required to attend is 75% of the total sessions. Each session will consist of: warm-up exercises for 5 min, CT, and cool-down for 5 min with global stretching exercises. Initially, participants will be asked for their perception of pain or discomfort through a visual analogue scale (VAS) and will be instructed to inform the location of the pain through the Nordic questionnaire musculoskeletal symptoms; baseline blood pressure (BPi) and initial heart rate (HRi) will also be measured. Subsequently, the participants will perform a warm-up exercise for 5 min on a stationary bicycle with minimum resistance.

**Fig. 2:**
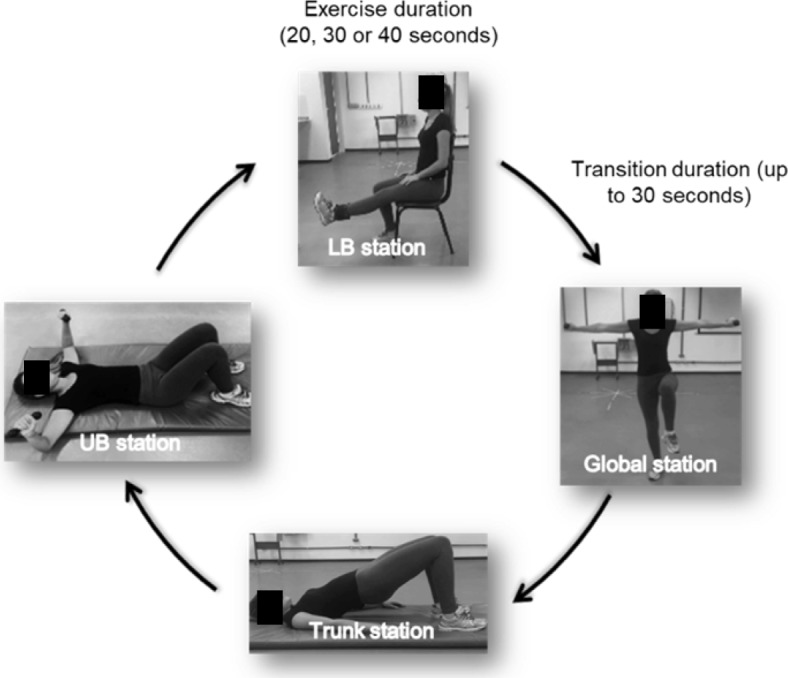
Circuit training protocol - Station organization

Immediately after the warm-up exercise, each participant will perform the circuit training protocol. The participants will be instructed to perform each exercise as fast as possible, with the maximum number of repetitions during the established time in each training phase (light, moderate, and intense). The first station of exercises to be performed will be defined in a random order at each session. After the end of the first exercise station, the participant will move to the next exercise station, following a specific order to allow different muscle groups to alternate between rest and work, favoring recovery and minimizing the risk of muscle fatigue [34] as shown in Fig. 2.

**Fig. 3:**
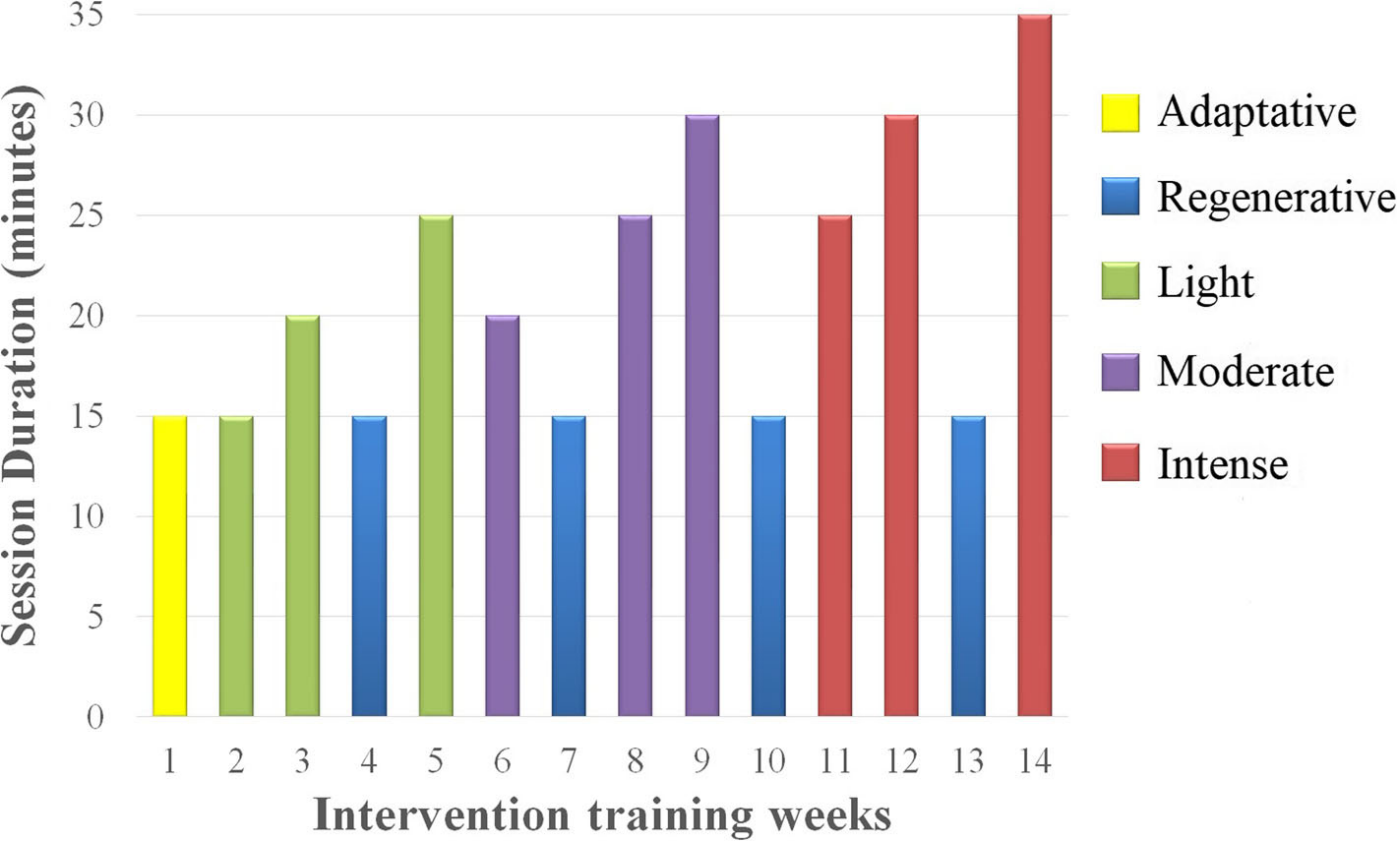
Periodized dynamic loads of the circuit training protocol

Periodized dynamic intensity is a strategy used to define the dynamic intensity according to weeks of training (Fig. 3). The volume of work is defined by the training session duration and through the previously stratified intensity of effort and heart rate response to exercise, as described above.

The construction of the periodization model to be used, regarding the biological principle of volume vs. intensity interdependence, and a duration of 14 weeks, proposes a week of recuperative exercises after two weeks of stress, gradually increasing the intensity with respective volume settings. This model is based on the concepts described by Turner (2011) which concludes in favor of the organization of training adapted to the reality of the public to be trained.

The representation in Fig. 3 can be detailed as follows: the yellow column represents the adaptation and familiarization week (1st week) in which participants will be familiarized with the materials and exercises performed during the training. The exercises previously stratified as light will be performed in the second, third, and fifth weeks of training (green columns), moderate exercises will be performed in the sixth, eighth, and ninth weeks (purple columns), and intense exercises will be performed in the eleventh, twelfth, and fourteenth weeks (red columns).

The blue columns represent regenerative weeks in which the training will be performed at light intensity, but in this case, each exercise will have a 10 s duration with an increase of 10 s in the recovery time of each station, accounting for a smaller workload with mild exercise.

The exercise intensity will be increased at the 6th and 11th weeks, as shown in Fig. 3. This will be based on the pilot study performed, as described.

It is possible to observe that from the second week, the protocol follows a progression method based on the intensity of effort and the volume of work (time). Three points must be observed in this model: i-) the three intensities, according to the proposed classification, are carried out for three weeks each; ii) every two weeks of training, with intensities that aim at adaptation through stress, there is a recovery week (increase of metabolism with less structural and cardiovascular impact) and; iii) the two weeks between the recoveries are composed of implementation in the workload or in the effort intensity.

Thus, the planning progresses in a nonlinear manner. Changes in volume and intensity offer new challenges every week and show how it becomes increasingly easy to perform lower-intensity exercise. The principle of adaptation suggests stress and recovery to achieve goals and this was the intention in setting up this routine: To cause a controlled stress (stratification already described), to increment such stress, (either in the workload or the intensity of the effort) and to alternate periods of increment and recovery to stimulate the perception of adaptation acquired by participants [84].

Immediately after each training session, RPE; Final blood pressure (BPf); final heart rate (HRf); and VAS will be measured again.

### Strength training protocol

Strength training is one of the most common types of exercise prescribed for the knee OA population, and in general has shown beneficial results on various parameters. For this reason, the strength training protocol used in this study will follow the model proposed by Selistre et al. (2017) with minor modifications. This protocol demonstrated reduced muscle weakness, pain, and disability in men with early knee osteoarthritis [81]. Each session will have an average duration of 60 min, with three sessions per week for 14 weeks, also totaling 42 exercise sessions. The same exercises as performed in the circuit training protocol were selected for the lower body and trunk. The strength training will also include the same warm-up (five minutes of stationary bicycle) and cool-down strategies (5 min global stretching).

Table 1 shows the exercise distribution during the 14-week strength training protocol. The strength training protocol is divided into three levels. In the first level (1st-5th weeks), three quadriceps strengthening exercises and one strengthening exercise for the hamstrings, hip abductors, and adductors will be performed. In the second level (6th–9th weeks), three quadriceps strengthening exercises and strengthening exercise for the hamstrings, hip abductors, and adductors, and three trunk exercises will be performed. In the third level (11th–14th weeks), the same exercises as in the second level will be performed, but with higher difficulty.

**Table 1:** 14-week Strength Training Protocol exercise distribution

Level/Weeks	Exercise
Level 1 - 1st to 5th weeks	Semi squats (50°)^a^
	Knee extensor strengthening with ankle weights.^a^
	Straight leg raises with ankle weights.^a^
	Hip abductor strengthening with ankle weights.^a^
	Hip adductor strengthening with ankle weights.^a^
	Knee flexor strengthening with ankle weights.^a^
Level 2 - 6th to 9th weeks	Level1
	Traditional bridge^a^
	Knee plank^b^
	Side knee plank^b^
Level 3 – 10th to 14th weeks	Level1
	Plank^b^
	Side plank^b^
	Bridge on bosu^b^

^a^ = *2 × 15 rep; 1 min between sets;*^b^ *= 3 × 10 seconds*

The initial load set for each exercise will be based on the one repetition maximum test (1 RM). Strengthening exercises will be performed in two sets of 15 repetitions, using 25% 1RM for hip adductors and abductors, and 50% 1RM for the quadriceps and hamstrings, using ankle weights. Ankle weights will be progressed if necessary. During knee extensor and flexor, and hip adductors and abductors strengthening, participants will perform each repetition with a 5 s isometric muscle contraction. Exercises for the trunk will be performed in three 10-s series, increasing the duration when participants are able.

### Educational protocol

The educational protocol will follow the model proposed by Messier et al. (2013b), in order to provide care, social interaction, and health education [2]. Meetings of 60 min will be held twice a month for 14 weeks, totaling 8 meetings. Interactive presentations will be performed addressing topics such as pathophysiology of osteoarthritis, and American College of Rheumatology (ACR) recommendations on nutrition, posture, and lifestyle. In addition, at the end of the meetings, participants will perform the same stretching exercise as performed in the exercised intervention protocols, but only on the upper body for five minutes, in order to improve adherence and increase the perceived benefits. According to the authors, there is no evidence that this practice of health education can directly influence the primary results of the present study [2]. Participants in the trained groups will also participate in the education and health sessions at separate times.

### Statistical analysis

Data analysis will be performed using the software *Statistical Package for the Social Sciences* version 20.0 (*SPSS* Inc., Chicago, IL, USA). An intention to treat analysis will be conducted for all data analyses. Initially, descriptive analysis of variables will be performed as mean, standard deviation, and coefficient of variation. Next, normality and homogeneity of variance will be checked by the Kolmogorov-Smirnov test and Levene’s test, respectively**.** If normal distribution of data is not observed, the same transformations will be carried out, to allow the application of parametric tests**.**

Two-way ANOVA (group X time) will be conducted for the dependent variables. When significant differences were observed in the group or interaction, will be performed a post-hoc Bonferroni Test. For all measured variables, the estimated sphericity will be examined in accordance with Mauchly’s W Test, and the Greenhouse-Geisser correction will be used as needed. For comparison between moments, for uCTX-II, sCOMP, and MVIC repeated measures ANOVA (baseline X during protocols X follow-up) will be performed. When significant main effects are found, the Bonferroni test will be used to identify statistical differences. For IL1-β, IL-6, TNF-α, leptin, adiponectin, WOMAC, PCS, DXA, computed tomography, and functional performance-based tests, the t-test will be used (baseline X follow-up). For all analyzes a significance level of 5% will be adopted (*p* < 0.05). No interim efficacy or subgroup analyses are planned.

## Discussion

Despite the large number of studies investigating the effects of physical exercise on the knee OA population, there are few clinical trials that focus on altered thigh composition (i.e. increased adipose tissue and decreased muscle mass). No studies were found which investigated the effects of a periodized circuit training protocol in this population, based on intensity of specific effort for this population with a randomized controlled trial model. As previously mentioned, circuit training promotes changes in body composition and muscle strengthening, as well as being a training modality tolerated by patients with knee OA, as attested in our pilot study. This training model has already been used in several other chronic dysfunctions and should be better explored considering its diverse benefits, both in skeletal muscle and the cardiorespiratory system.

In addition to the physical benefits, it is worth emphasizing the financial benefits that this modality provides, considering the greater number of participants in the same session, shorter session duration, and the possibility of performing the exercises without needing machines, commonly used in strength training.

Given the lack of information in the published literature and the public health impact that exercise intervention protocols can provide, the effects of periodized circuit training on thigh composition (adipose tissue and muscle mass), pain, functional performance, inflammation, and degeneration biomarkers are critical to delineate.

This study follows the OARSI recommendations for designing clinical trials for patients with knee OA [48] as well as the CONSORT recommendations for performing randomized controlled trials [54]. In addition, this study also has as a differential a control group, based on the model proposed by Messier et al. 2013. This educational protocol comparison group provides attention, social interaction, and health education. The purpose of including this group is to encourage recruitment, adherence, and benefit, and not to influence the primary outcome directly: no evidence was found that suggests that health education alone can affect body composition, pain, muscle strength, functional performance, or biomarkers. Considering that older adults are less likely to attend if they think any treatment group does not provide personal benefit [2, 45], including a control group providing similar benefits, such as those typically offered by community health-education programs, seems to be a good way to improve the feasibility of the study.

Thus, considering the high prevalence and various disorders related to knee OA, as well as the gaps in the scientific literature about the ideal prescription of exercise training protocols, assessing the efficacy of a periodized circuit training protocol, with a design focused on intensity progression, and its impact on body composition, as well as on the various related outcomes, has immediate and high clinical impact. The results of this study will provide critically needed guidance to the health care system for the treatment and prevention of complications related to knee OA.

## Additional file

Additional file 1: Circuit Training Protocol – Selected exercises descriptions. (DOCX 5584 kb)
