# Supplementary material for: Influence of a periodized circuit training protocol on intermuscular adipose tissue of patients with knee osteoarthritis: protocol for a randomized controlled trial
Source: BMC Musculoskelet Disord. 2018 Nov 30;19:421. doi: 10.1186/s12891-018-2325-y (PMC6267088; doi:10.1186/s12891-018-2325-y)
Supplement: Supplementary file 1 — Circuit Training Protocol – Selected exercises descriptions. (DOCX 5584 kb) [file 12891_2018_2325_MOESM1_ESM.docx]

**Circuit Training Protocol – Selected exercises descriptions**

**Mild Exercises -From 2^nd^ to a 5^th^ week**

| **Upper body exercises** | |
| --- | --- |
| 1.Supine position - Shoulder abduction with elbow flexion holding dumbbells | 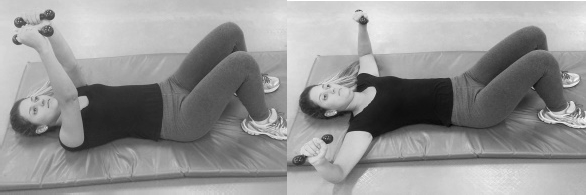 |
| 2. Standing position- Alternating punches without weight | 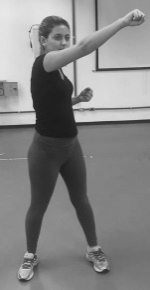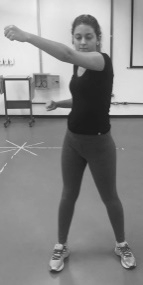 |
| 3. Standing position- Elbow flexion with 0.5 kg dumbbell | 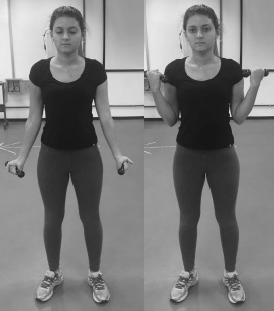 |
| **Lower body exercises** | |
| 4. Sitting position – knee flexion light elastic band | 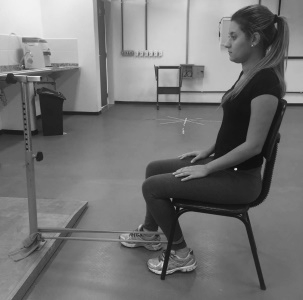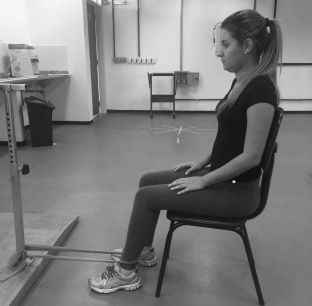 |
| 5. Standing position- Step-up and down | 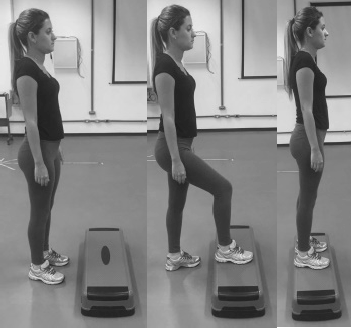 |
| 6. Standing position- Hip Abduction with 0.5kg ankle weight | 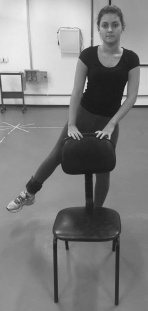 |
| 7. Standing position – Calf strengthening without extra weight | 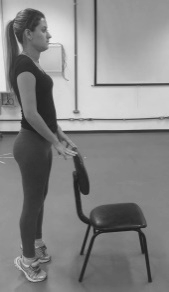 |
| 8. Supine position – Single leg raises with 0.5kg ankle weight | 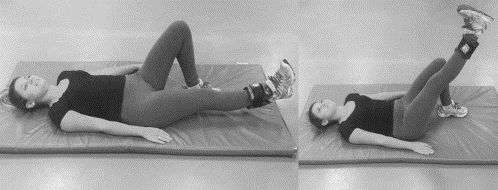 |
| 9. Supine position- Traditional Bridge | 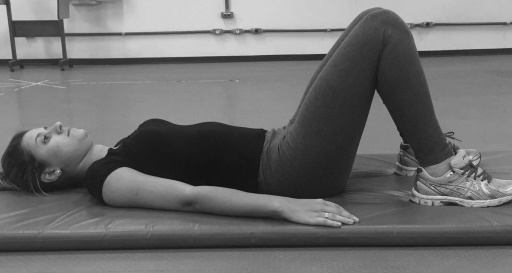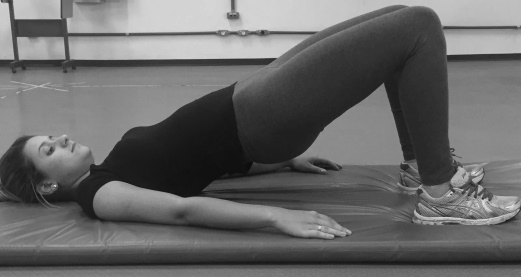 |
| **Trunk exercises** | |
| 10. Ventral position – Knee Plank (isometric) | 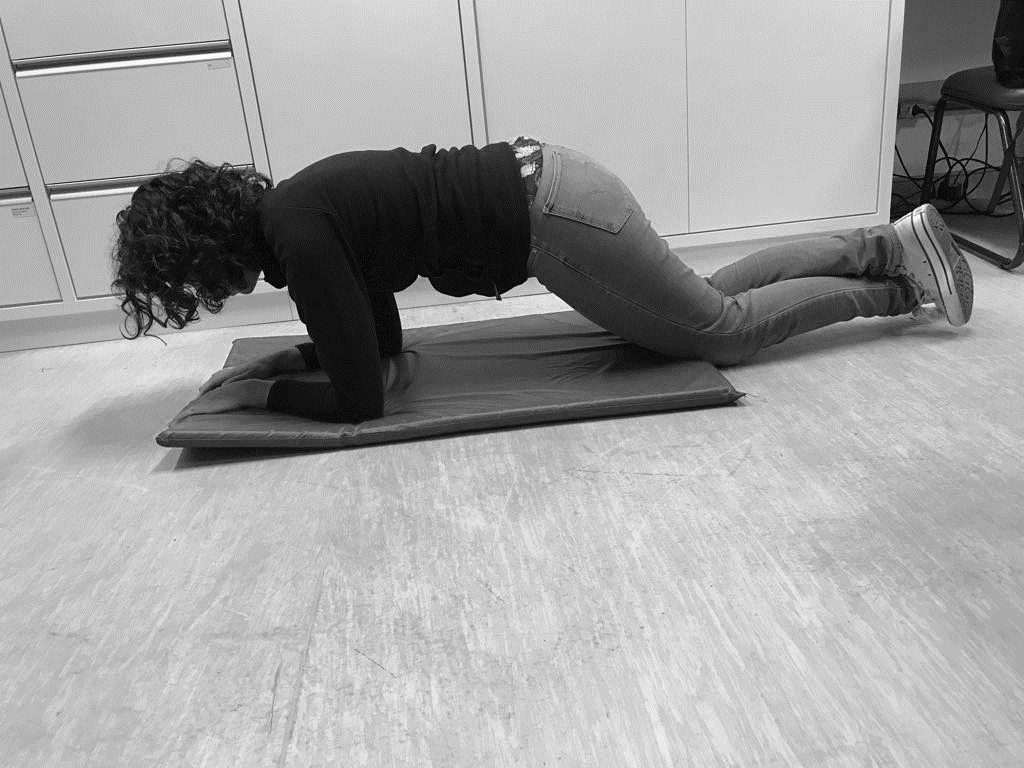 |
| 11. Knee Side Plank (isometric) | 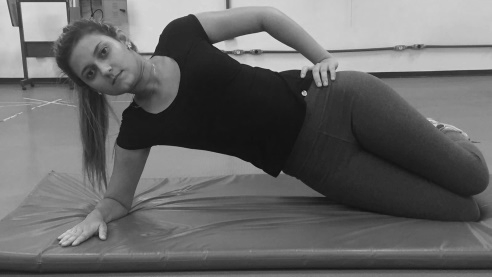 |
| 12. Supine position - Abdominal curl with legs leaning on the ball | 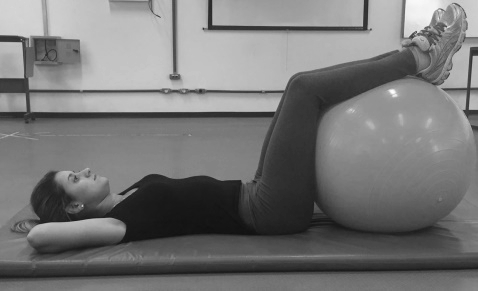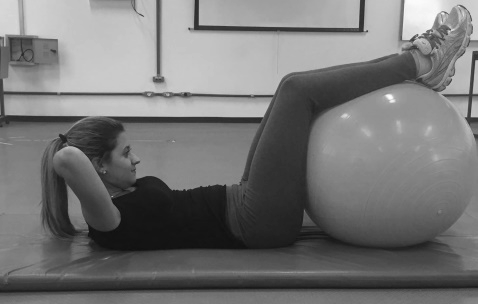 |
| **Global exercise** | |
| 13. Standing position - Elbow flexion with 0.5kg dumbbell associated with hip and knee flexion contraleteral | 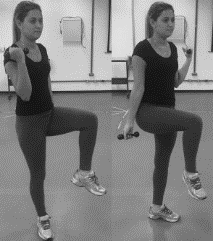 |
| 14. Standing position – Shoulders abduction with 0.5kg dumbbell associated with hip flexion | 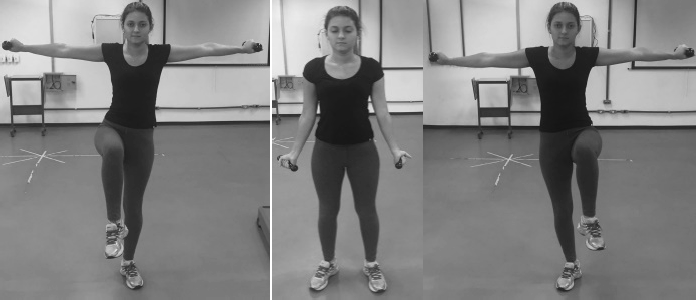 |
| 15. Standing position -Jumping Jack adapted (no jumping): starting with arms extended over the head and legs adducted. Progress the exercise to shoulders 90^o^ abduction and legs abduction. | 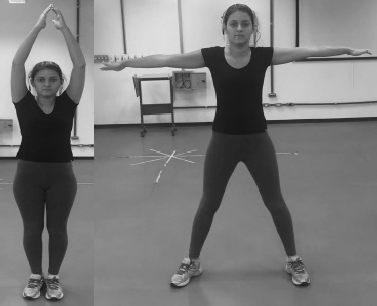 |

# Moderate Exercises- From 6^th^ to 10^th^ week

| **Upper body exercises** | |
| --- | --- |
| 1. Standing position- Alternating punches | With 0.5kg dumbbell |
| 1. Standing position – Elbow flexion | With 0.5kg dumbbell |
| 1. Standing position – Shoulder flexion with light elastic band | 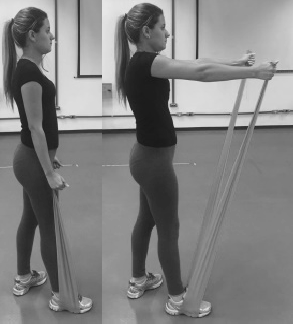 |
| 1. Standing position – Elbow flexion with light elastic band | 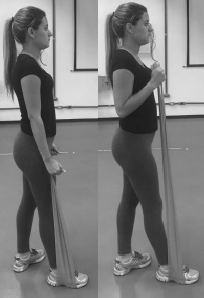 |
| **Lower body exercises** | |
| 1. Standing position- Hip Abduction with light elastic band | 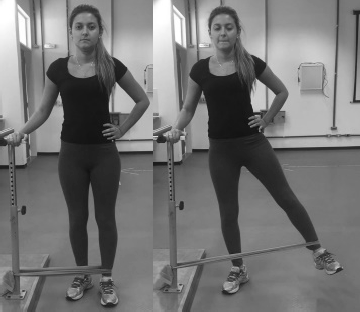 |
| 1. Stand up position – Hip adduction with light elastic band | 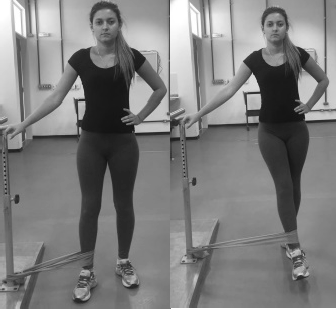 |
| 1. Sit-to-stand | 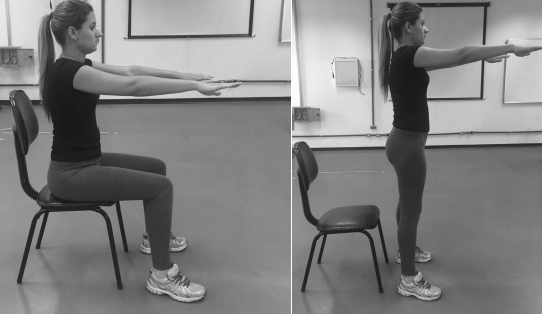 |
| 1. Siting position – Knee extension with 0.5kg ankle weight | 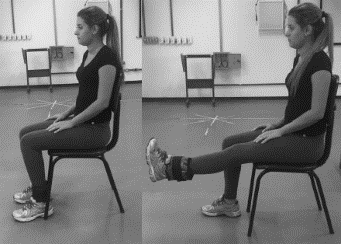 |
| 1. Standing position – Knee flexion with 0.5kg ankle weight | 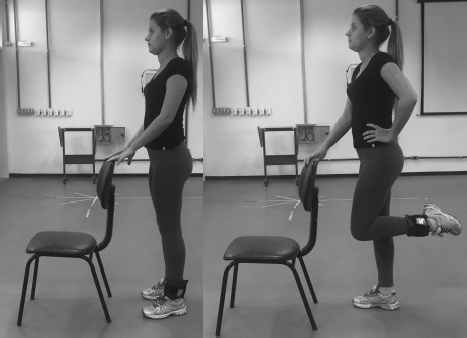 |
| **Trunk exercises** | |
| 1. Supine position – Abdominal curl up holding a small ball | 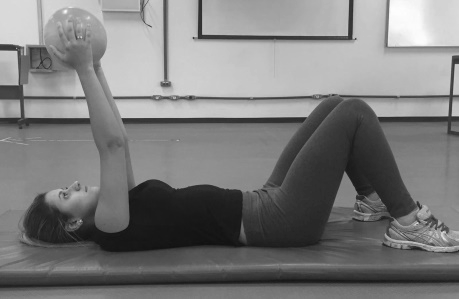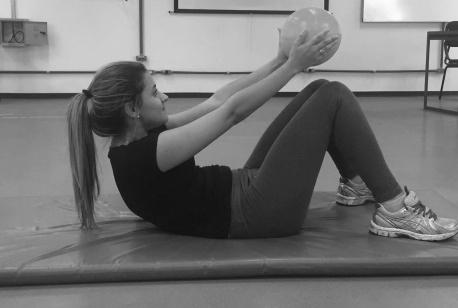 |
| 1. Supine position – bridge exercise with hip adduction holding a small ball between the knees | 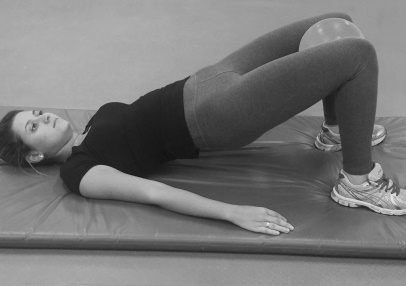 |
| 1. Plank (isometric) | 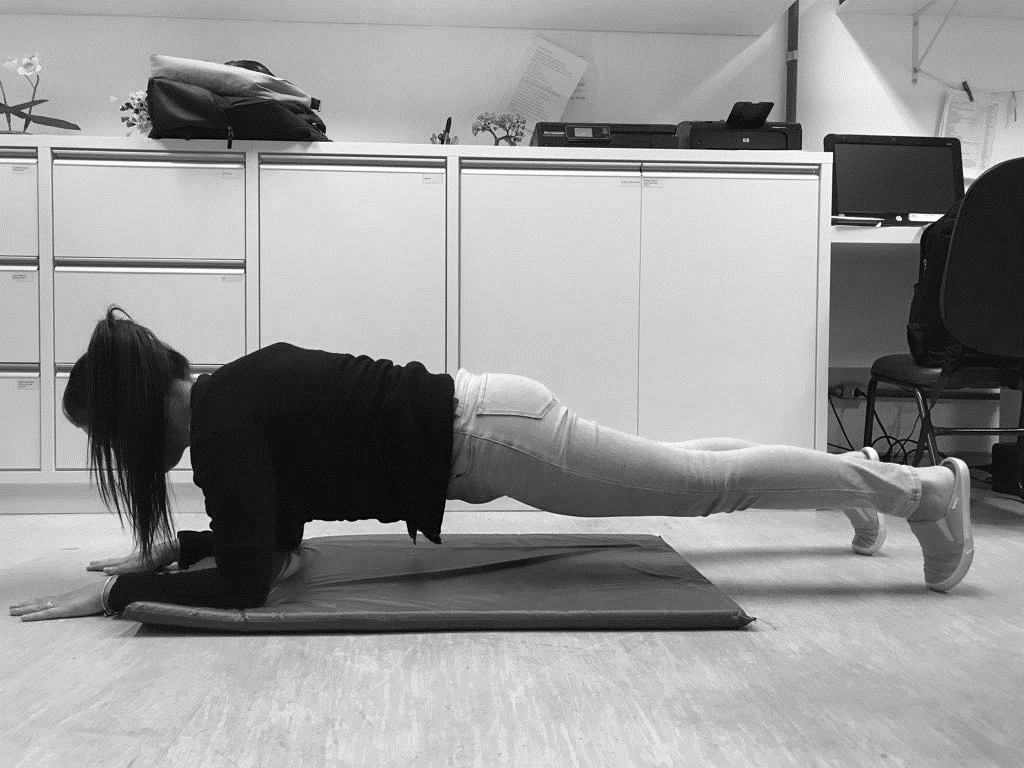 |
| 1. Side Plank (isometric) | 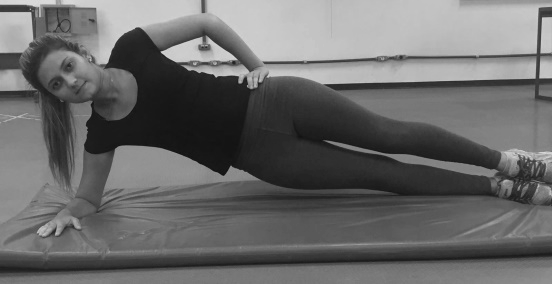 |
| **Global Exercises** | |
| 1. Standing position - Elbow flexion with 1.0kg dumbbell associated with hip and knee flexion contralateral | 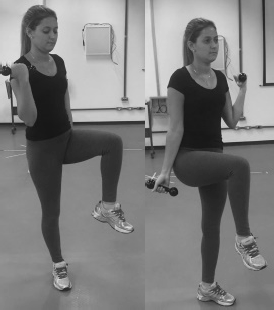 |
| 1. Standing position – Shoulders abduction with 1.0kg dumbbell associated with hip and knee flexion | 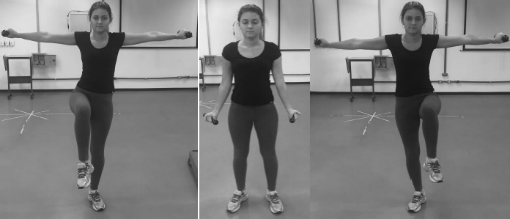 |
| 1. Standing position -Jumping Jack adapted (no jumping) with 0.5kg dumbbell | 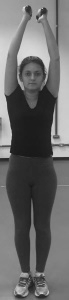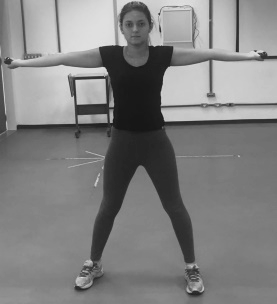 |
| 1. Squat holding a small ball | 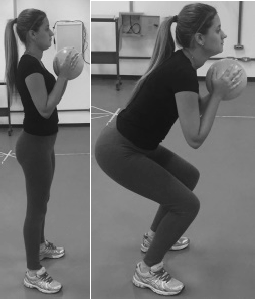 |

**Intense Exercises - From 11^th^ to 14^th^ week**

| **Upper body exercises** | |
| --- | --- |
| 1. Standing position- Alternating punches with 1.0kg dumbbell | With 1.0kg dumbbell |
| 1. Standing position – Shoulder flexion | With hard elastic band |
| 1. Standing position – Elbow flexion | With hard elastic band |
| **Lower body exercises** | |
| 1. Standing position- Hip Abduction | With hard elastic band |
| 1. Stand up position – Hip adduction | With hard elastic band |
| 1. Standing position – Knee flexion | With 1.0 kg ankle weight |
| 1. Forward Lunge with elastic band medially traction. | 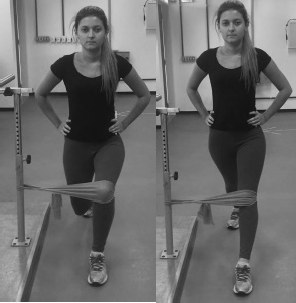 |
| **Trunk exercises** | |
| 1. Supine position – bridge with single leg static hold - one foot on the floor and contralateral limb with knee extension | 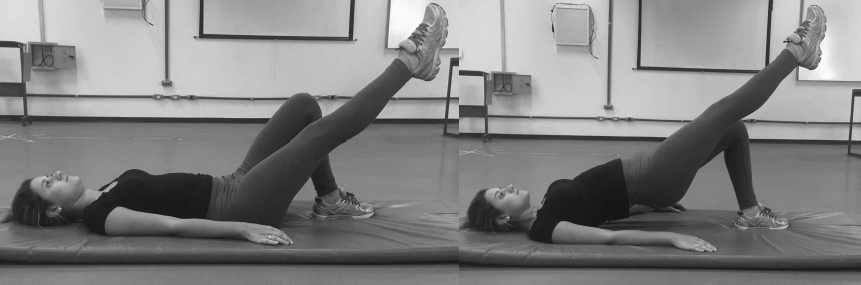 |
| 1. Supine position – Oblique abdominal strengthening holding a small ball | 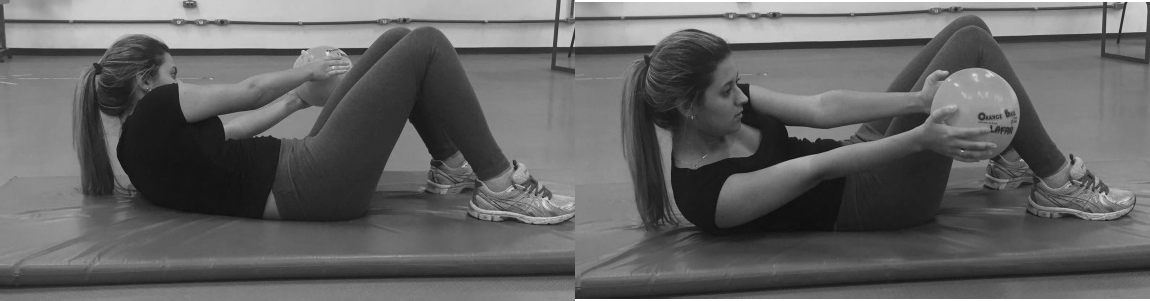 |
| 1. Supine position – Bridge on bosu | 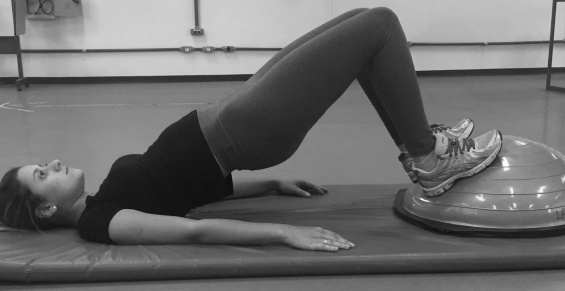 |
| 1. Supine position – Abdominal curl with hip flexion and knee extension holding a small ball | 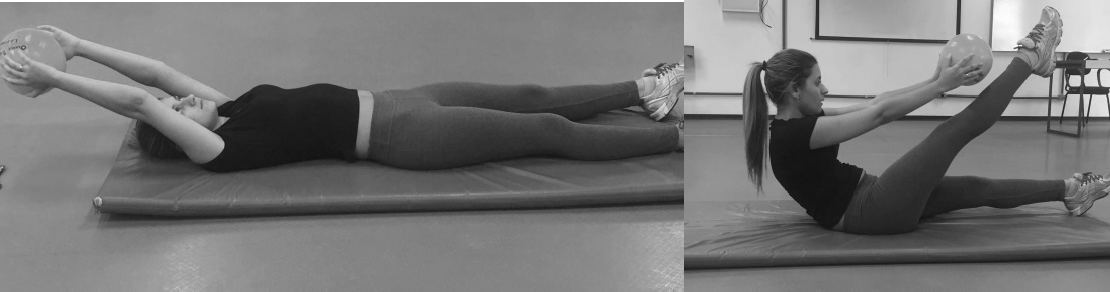 |
| **Global exercises** | |
| 1. Standing position – jumping Jack adapted (no jump) | With 1.0 kg dumbbell |
| 1. Standing position – Trunk flexion with hands touching the contralateral ankle (knee bended) | 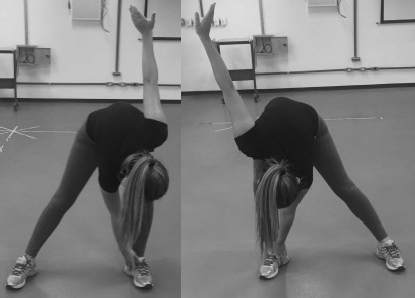 |
| 1. Standing position – Criss cross with 1.0kg dumbbell | 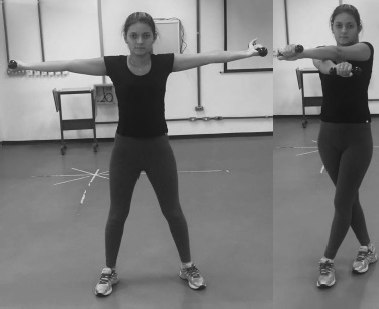 |
| 1. Standing position – squat to overhead press with 0.5kg dumbbell | 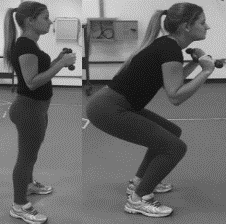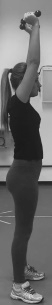 |
